# Comparative Optimization of Microwave and Ultrasound-Assisted Extraction of Bioactive Compounds and Proteins from *Craterellus cornucopioides*

**DOI:** 10.3390/jof12030215

**Published:** 2026-03-17

**Authors:** Mojca Čakić Semenčić, Filip Šupljika, Anet Režek Jambrak, Monika Kovačević, Nina Čuljak, Ivana Repić, Ksenija Markov, Jadranka Frece

**Affiliations:** 1 Department of Chemistry and Biochemistry, Faculty of Food Technology and Biotechnology, University of Zagreb, Pierottijeva 6, 10000 Zagreb, Croatia; filip.supljika@pbf.unizg.hr (F.Š.); monika.kovacevic@pbf.unizg.hr (M.K.); 2 Laboratory for Sustainable Development, Faculty of Food Technology and Biotechnology, University of Zagreb, Pierottijeva 6, 10000 Zagreb, Croatia; anet.rezek.jambrak@pbf.unizg.hr; 3 Laboratory for General Microbiology and Food Microbiology, Faculty of Food Technology and Biotechnology, University of Zagreb, Pierottijeva 6, 10000 Zagreb, Croatia; nina.culjak@pbf.unizg.hr (N.Č.); ivana.repic@pbf.unizg.hr (I.R.); ksenija.markov@pbf.unizg.hr (K.M.); jadranka.frece@pbf.unizg.hr (J.F.)

**Keywords:** *Craterellus cornucopioides*, microwave-assisted extraction, ultrasound-assisted extraction, bioactive compounds, extraction optimization, antioxidant potential, antimicrobial activity

## Abstract

This study investigates the optimization of extraction processes for bioactive compounds and proteins from the mushroom *Craterellus cornucopioides* by comparing Microwave-Assisted Extraction and Ultrasound-Assisted Extraction. Using Response Surface Methodology, the effects of temperature or amplitude, time, and solvent type were evaluated on total phenols, flavonoids, proteins, glutathione content, and antioxidant capacity measured by DPPH and FRAP assays. Additionally, the antimicrobial potential of the extracts was screened against various pathogens. Results demonstrated that water was the most effective solvent for nearly all parameters across both techniques, providing a unified optimum in the ultrasound system at six minutes and one hundred percent amplitude. However, a notable exception was observed for glutathione recovery in the microwave system, where ethanol proved superior to water. Ultrasound-assisted extraction consistently outperformed microwave extraction in protein yield and overall antioxidant potential, offering a more robust approach regarding process efficiency and bioactive yield. In conclusion, while both green techniques enhance recovery, ultrasound extraction with water establishes itself as the most consistent method for the simultaneous extraction of bioactives.

## 1. Introduction

Edible and medicinal mushrooms are increasingly recognized as high-value, sustainable resources for the food, pharmaceutical, and biotechnological industries due to their diverse bioactive profiles [1,2,3,4]. Among these, higher fungi serve as natural reservoirs of polysaccharides, proteins, sterols, and phenolic acids, contributing to documented antioxidant, antimicrobial, and anti-inflammatory activities [5]. Specifically, the Black Trumpet (*Craterellus cornucopioides*), also known as the “horn of plenty” or “black chanterelle,” is an ectomycorrhizal mushroom highly valued in gastronomy, where it is often dried and ground as a flavor enhancer known as “the poor man’s truffle” [6]. Despite growing exclusively in the wild, its nutritional merits—characterized by high water content, low fat, and a significant energy value (248.0–413.2 kcal/100 g dry matter)—underscore its potential as a functional food source [7,8]. *C. cornucopioides* is particularly notable for its exceptionally high total soluble protein content (up to 126.6 mg/g) and a complex array of secondary metabolites [9,10]. Phenolic compounds, including phenolic acids and flavonoids, are primarily responsible for the mushroom’s immunomodulatory and antimicrobial effects, with extraction yields often significantly influenced by solvent polarity [11]. Furthermore, the lipid fraction is dominated by ergosterol and monounsaturated fatty acids (61.4%), primarily oleic acid, while linoleic acid is the most prominent polyunsaturated fatty acid present in the profile [12]. Beyond macronutrients, this species contains significant levels of vitamin B_12_ and an unusually high concentration of manganese compared to other edible mushrooms [13].

The efficient and consistent recovery of these valuable compounds from the complex lignocellulosic matrix of the mushroom is crucial. Traditional solid–liquid extraction methods are often lengthy and risk degrading thermolabile compounds, thereby potentially compromising the stability and potency of the bioactive molecules. Therefore, modern, intensified techniques such as Ultrasound-Assisted Extraction (UAE) and Microwave-Assisted Extraction (MAE) are increasingly favored due to their ability to reduce extraction time, lower solvent usage, and improve overall yields. The primary objective of this study is to optimize and compare the efficiency of MAE and UAE for the extraction of bioactive substances from *C. cornucopioides* using Response Surface Methodology (RSM). Specifically, we determined and compared the yield of total polyphenols, total flavonoids, and proteins, and subsequently evaluated the correlation between these yields and the assessed antioxidant potential and antimicrobial activity.

## 2. Materials and Methods

### 2.1. Chemicals, Plant Material and Microorganisms

Most chemicals were purchased from Sigma-Aldrich (St. Louis, MO, USA). Other chemicals and reagents were obtained from various manufacturers as follows: sodium hydroxide, potassium dihydrogen phosphate, dipotassium hydrogen phosphate, and potassium sodium (K-Na) tartrate from Lach-Ner (Neratovice, Czech Republic); anhydrous sodium carbonate and 96% ethanol from KT.T.T. (Sveta Nedjelja, Croatia); and 37% hydrochloric acid from Kemika (Zagreb, Croatia). Gallic acid was supplied by Acros Organics (Fair Lawn, NJ, USA), while iron(III) chloride was sourced from GRAM-MOL (Zagreb, Croatia). Sodium acetate trihydrate was purchased from Lach-Ner s.r.o. (Neratovice, Czech Republic), and glacial acetic acid was obtained from Alkaloid AD Skopje (Skopje, North Macedonia).

Dried Black Trumpet mushrooms were provided by VG FRYER d.o.o. (Veliki Grđevac, Croatia). Prior to extraction, the mushrooms were ground using a Retsch GM 200 mill to a particle size ranging from 500 to 1000 µm, which corresponds to a 35 to 18 mesh size.

Test microorganisms used in this study were obtained from the ATCC (American Type Culture Collection) microbial repository and are part of the microbial collection of the Laboratory for General Microbiology and Food Microbiology at the University of Zagreb, Faculty of Food Technology and Biotechnology. The test microorganisms included the bacteria *Proteus mirabilis* ATCC^®^ 25933^TM^, *Escherichia coli* ATCC^®^ 25922^TM^, *Bacillus subtilis* ATCC^®^ 6633^TM^, *Pseudomonas aeruginosa* ATCC^®^ 27853^TM^, *Staphylococcus aureus* ATCC^®^ 25923^TM^, *Salmonella typhimurium* ATCC^®^ 29631^TM^ and *Listeria monocytogenes* ATCC^®^ 23074^TM^, as well as the yeast *Candida albicans* ATCC^®^ 10231™. All bacterial cultures were stored at −80 °C in nutrient broth (Biolife, Milan, Italy), while the yeast was stored in malt broth (Biolife, Milan, Italy) supplemented with 15% glycerol (GRAM-MOL, Zagreb, Croatia).

### 2.2. Extractions

MAE was carried out using a Milestone Start S microwave reactor (Milestone, Sorisole, Italy). For each experimental run, 3 ± 0.001 g of crushed mushroom sample was mixed with 100 mL of deionised water in a round-bottom flask and homogenized. The flasks were fitted with magnetic stirrers to ensure uniform heating. The microwave power was set at 300 W, while the temperature (40 °C or 60 °C) and exposure time (3 or 6 min) were varied according to the experimental design (Table 1). UAE was performed using a Q700 probe-type sonicator (Qsonica, Newtown, CT, USA) with a 19 mm diameter probe. As in the MAE procedure, 3 ± 0.001 g of the sample was suspended in 100 mL of deionised water and homogenized. The probe was immersed directly into the suspension, ensuring that it did not touch the beaker walls. A thermocouple was used to monitor the temperature in real time. Sonication was conducted at amplitudes of 50% or 100% for 3 or 6 min (Table 1). To prevent thermal degradation from overheating, the sample beaker was placed in an ice bath throughout the process.

Following extraction, the samples were filtered using a Büchner funnel. The pH of the extracts was then measured with a 913 pH meter (Metrohm AG, Herisau, Switzerland), and the electrical conductivity was determined using a Cond 315i conductivity meter (WTW, Weilheim, Germany).

### 2.3. Labeling of Samples

The samples in Table 1 are labeled based on the extraction method and the solvent used. The first letter refers to the extraction technique: M denotes MAE, while U represents UAE. The second letter indicates the solvent used for the process: W stands for deionized water, and E refers to 30% ethanol.

### 2.4. Analysis

Unless otherwise specified, all analyses were performed using the crude, undiluted extracts obtained after filtration. Measurements were conducted in duplicate, or in triplicate if the difference between measurements exceeded 10%. To ensure consistency across all bioactive and antioxidant assays, results were calculated based on the standardized dry matter content of the mushroom powder (99.18% ± 0.05%), which was determined by drying the samples to a constant weight at 105 °C according to the literature method [14]. All final values are expressed per gram of dry matter (mg/g d.m.).

#### 2.4.1. Determination of Total Phenolic and Total Flavonoid Content

The evaluation of the bioactive potential and antioxidant capacity was performed through the determination of total phenolic (TPC) and flavonoid (TFC) contents. This approach aligns with established protocols for assessing the chemical composition and biological properties of plant and fungal extracts, as demonstrated by Shi et al. [15], who utilized UHPLC-Q-Exactive Orbitrap Mass Spectrometry to provide a comprehensive profile of phenolic acids and flavonoids linked to their antioxidant and anti-inflammatory activities. The TPC was determined using a modified Folin–Ciocalteu assay [16]. Briefly, 100 µL of the extract was mixed with 200 µL of Folin–Ciocalteu reagent and 2 mL of distilled water in a glass test tube. After 3 min, 1 mL of a 20% sodium carbonate solution was added. The mixture was vortexed and incubated at 50 °C for 25 min. After cooling, the absorbance of the resulting blue complex was measured at 765 nm. A blank was prepared following the same procedure by replacing the extract with the extraction solvent. TPC was calculated using a gallic acid standard curve:

(1) *y* = 0.00345*x* + 0.03097

where “*y*” is the absorbance at 765 nm and “*x*” is the gallic acid concentration (µg/mL). Results are expressed as mg of gallic acid equivalents per gram of dry mass (mg GAE/g d.m.).

TFC was assessed using an aluminum chloride colorimetric method, based on the procedure by Chang et al. [17] with modifications. The reaction mixture contained 0.5 mL of extract, 1.5 mL of 96% ethanol, 0.1 mL of 10% aluminum chloride, 0.1 mL of 1 M potassium acetate, and 2.8 mL of distilled water. After incubation in the dark for 30 min, absorbance was measured at 415 nm. TFC was quantified using a quercetin standard curve:

(2) *y* = 0.0057*x* + 0.01670

where “*y*” is the absorbance at 415 nm and “*x*” is the quercetin concentration (µg/mL). Results are expressed as mg of quercetin equivalents per gram of dry mass (mg QE/g d.m.).

#### 2.4.2. Determination of Total Protein Content

The total protein concentration in the extracts was quantified using two distinct colorimetric assays: the Bradford method [18] and the Lowry method [19], with bovine serum albumin (BSA) as the reference standard. Reagent C was prepared by mixing Reagent A (2% Na_2_CO_3_ in 0.1 M NaOH) and Reagent B (0.5% CuSO_4_·5H_2_O in 1% sodium potassium tartrate) in a 50:1 ratio (*v*/*v*). A 0.4 mL aliquot of a five-fold diluted extract was mixed with 2 mL of this reagent and homogenized. Following an initial incubation of 10–15 min at room temperature, 0.2 mL of Folin–Ciocalteu reagent (diluted 1:2 with deionized water) was rapidly added, and the mixture was vortexed vigorously. The resulting solution was then allowed to stand at room temperature for 40–60 min to achieve full color development. The absorbance was measured at 740 nm. Protein concentration was calculated using the linear equation:

(3) *y* = 0.002*x* + 0.0457

where “*y*” represents a measured absorbance at 740 nm and “*x*” repsents BSA equivalents (in μg/mL).

For the Bradford assay, 0.4 mL of a five-fold diluted extract was mixed with 1.6 mL of commercial Bradford reagent. The mixture was incubated in the dark at room temperature for 10 min. Absorbance was then recorded at 595 nm, and protein concentration was determined using the linear equation:

(4) *y* = 0.0068*x* + 0.027

where “*y*” represents a measured absorbance at 595 nm and “*x*” BSA equivalents (in μg/mL).

The results for both assays are expressed as mg of protein per gram of dry mass (mg/g d.m.).

#### 2.4.3. Determination of Protein Extracts by SDS-PAGE

To determine the protein profile of mushroom extracts, the extracts were subjected to SDS-PAGE according to Laemmli [20], with slight modifications. Extracts were centrifuged at 13 000 rpm for 15 min to remove insoluble debris and to obtain a supernatant suitable for electrophoretic analysis. Subsequently, 40 µL of the supernatant was mixed with 10 µL of 2× Laemmli buffer (1.25 mL 1 M Tris–HCl, pH 6.8; 4 mL 10% (*w*/*v*) SDS; 2 mL glycerol (100%, *v*/*v*); 0.5 mL 0.5 M EDTA; 4 mg bromophenol blue; and 0.2 mL β-mercaptoethanol), boiled for 5 min, and 20 µL of each sample was loaded onto 12% (*v*/*v*) polyacrylamide gel. Protein separation was carried out by SDS–PAGE in an electrophoresis chamber at a constant voltage of 160 V for 1.5 h. The Broad Range Protein Marker 6.5–212 kDa (New England Biolabs, Ipswich, MA, USA) was used as a molecular weight standard. Following electrophoresis, the gel was incubated in a staining solution (0.02% Coomassie Brilliant Blue, 25% isopropanol, and 10% acetic acid) for 2 h and subsequently destained in 7% (*v*/*v*) acetic acid until the background became clear. Controls consisted of samples prepared using 30% ethanol and distilled water instead of the mushroom extract.

#### 2.4.4. Determination of the Antioxidant Activity Using DPPH and FRAP Methods

The DPPH radical scavenging activity (RSA) was determined using a modified literature method [21]. A 0.101 mM DPPH working solution was prepared by dissolving 4 mg of DPPH in 100 mL of 96% ethanol; the solution had an initial absorbance of approximately 1.0 at 517 nm. For the assay, 100 µL of extract was added to 2.5 mL of the DPPH solution. The mixture was shaken and incubated in the dark for 30 min. Absorbance was then measured at 517 nm. A control sample was prepared by replacing the extract with the extraction solvent. The RSA percentage was calculated using the following formula:

(5) $$\mathrm{RSA}\;(\%)\;=\;\left(\frac{Ac\;-\;As}{Ac}\right)\times 100$$

where *A*c is the absorbance of the control sample and *A*s is the absorbance of the test sample. Results are expressed as mean percentage RSA ± standard deviation.

The ferric reducing antioxidant power (FRAP) was determined according to the method of Shortle et al. [16], based on the reduction of the ferric tripyridyltriazine complex to its blue ferrous form. The FRAP reagent was prepared by mixing 0.3 M acetate buffer (pH 3.6), 10 mM TPTZ solution (in 40 mM HCl), and 20 mM FeCl_3_·6H_2_O in a 10:1:1 (*v*/*v*/*v*) ratio, followed by incubation at 37 °C for 10 min. Quantification was performed using an ascorbic acid calibration curve, yielding the linear regression equation:

(6) *y* = 2.0068*x* + 0.1091

where “*y*” represents a measured absorbance at 593 nm and “*x*” denotes the ascorbic acid equivalent (AAE) expressed in μmol/L. The FRAP values were calculated based on the sample mass and dry matter content and are expressed as mg of ascorbic acid equivalents per gram of dry mass (mg AAE/g d.m.).

#### 2.4.5. Determination of the Antioxidant Activity Using Ellman’s DTNB Assay

Antioxidant activity was determined using a modified method based on Ellman’s reagent (DTNB), described by Jakopović et al. [22]. The method is based on the reaction between glutathione (GSH) and 5,5′-dithiobis-(2-nitrobenzoic acid) (DTNB), in which the thiol group of GSH cleaves the disulfide bond of DTNB, resulting in the formation of the yellow-colored 5-thio-2-nitrobenzoate (TNB^−^) anion. The intensity of the developed color, measured spectrophotometrically at 412 nm, is proportional to the concentration of thiol-containing compounds present in the sample [23]. In a 96-well microtiter plate, 170 µL of 1 M K-phosphate buffer (pH 7.4), 10 µL of extract, and 20 µL of 1 mM DTNB were added to each well. For controls, distilled water or 30% ethanol was added instead of the extracts. The plate was incubated in the dark for 10 min, and the intensity of the resulting colored GSH-DTNB complex was monitored by measuring absorbance at 412 nm using a UV-Vis spectrophotometer (PerkinElmer, Waltham, MA, USA). The concentration of reduced glutathione (GSH) (µM) was calculated as follows:

(7) $$c\;(\mathrm{GSH})\;=\;\frac{A}{\varepsilon \cdot l}$$

where *A* is the measured absorbance at 412 nm, *Ɛ* is the molar absorption coefficient (14.15 mM^−1^ cm^−1^) and *l* is the path length of light through the solution (0.62 cm). The final values are expressed in µmol GSH per gram of dry weight (µmol GSH/g d.m.).

#### 2.4.6. Determination of Antimicrobial Activity

Antimicrobial activity was assessed using disk diffusion, agar well diffusion and turbidimetric assays. All methods employed the same test microorganisms—*P. mirabilis* ATCC^®^ 25933^TM^, *E. coli* ATCC^®^ 25922^TM^, *B. subtilis* ATCC^®^ 6633^TM^, *P. aeruginosa* ATCC^®^ 27853^TM^, *S. aureus* ATCC^®^ 25923^TM^, *S. typhimurium* ATCC^®^ 29631^TM^, *L. monocytogenes* ATCC^®^ 23074^TM^, and *C. albicans* ATCC^®^ 10231™. Cell density of the test microorganisms was standardized to 0.2 McFarland (≈10^6^ CFU/mL).

##### Disk Diffusion Assay

The disk diffusion method was performed according to Dogra et al. (2022) [24] with slight modifications. A total of 100 µL of the test microorganism was inoculated onto nutrient agar (for bacteria) or malt agar (for yeast) plates. Sterile disks (6 mm in diameter; Macherey-Nagel GmbH, Gutenberg, Germany) were sterilely immersed in the extracts, allowed to absorb the extract, and placed onto the inoculated agar plates. Disks soaked in 30% ethanol and distilled water were used as controls. Following incubation under aerobic conditions at 37 °C for bacteria or 28 °C for yeast for 48 h, the inhibition zones were measured.

##### Agar Well Diffusion Assay

The agar well diffusion assay was determined according to Čuljak et al. [25] with minor modifications. Nutrient agar (for bacteria) or malt agar (for yeast) plates were inoculated with 1 mL of the test microorganism. Wells with a diameter of 7 mm were punched into the agar with a borer, and 150 µL of the extract was added to each well. Control included wells containing 150 µL of 30% ethanol or distilled water. Following incubation in aerobic conditions at 37 °C for bacteria or 28 °C for yeast for 48 h, the inhibition zones were measured.

##### Turbidimetric Assay

The turbidimetric assay was performed according to Othman et al. [26] with minor changes. In a microtiter plate, 180 µL of each extract and 20 µL of the test microorganism were added to the wells. Additional sample setup included 90 µL of nutrient or malt broth, 90 µL of the test microorganism, and 20 µL of the test microorganism. The controls included: 180 µL of broth + 20 µL of the test microorganism; 180 µL of water or 30% ethanol + 20 µL of the test microorganism; and 90 µL of nutrient or malt broth + 90 µL of water or 30% ethanol + 20 µL of the test microorganism. Plates were incubated under aerobic conditions at 37 °C for bacteria and 28 °C for yeast. During the incubation period, the absorbance at 620 nm was measured using a microplate reader (Tecan, Männedorf, Switzerland) at 0, 2, 4, and 24 h.

#### 2.4.7. Experimental Design and Statistical Analysis

The experimental design and statistical analysis for both MAE and UAE were performed using STATGRAPHICS Centurion 19 (StatPoint Technologies, Inc., Warrenton, VA, USA). A two-level factorial design (2^3^) was implemented for each method to systematically evaluate the influence of key independent variables on the extraction yields of bioactive compounds. For MAE, the independent variables were as follows: treatment time (TT: 3 and 6 min), temperature (T: 40 and 60 °C), and solvent type (S: water and 30% ethanol). For UAE, the independent variables were as follows: treatment time (TT: 3 and 6 min), ultrasound amplitude (A: 50% and 100%), and solvent type (S: water and 30% ethanol). The selection of these parameters and their ranges was based on preliminary tests. The dependent variables (responses) included TPC, TFC, protein content (by Bradford and Lowry methods), glutathione content (determined by Ellman’s assay) and antioxidant activity (DPPH and FRAP assays). A multifactor analysis of variance was first performed to determine the statistical significance (*p* < 0.05) of the main effects of each independent variable within a 95.0% confidence interval. Subsequently, Response Surface Methodology (RSM) was applied to model the interactions between factors and to predict optimal extraction conditions. For the RSM analysis, a full quadratic model with all two-factor interactions was fitted. While these models showed excellent predictive power (*R*^2^ > 0.97 for most responses), the limited degrees of freedom inherent to the saturated factorial design prevented valid significance testing of individual interaction terms via *F*-tests. Therefore, the statistical significance of the main factors was definitively assessed using a main-effects-only multifactor analysis of variance model, while the RSM models were utilized for their predictive capability in optimization and for interpreting factor interactions.

The primary optimization goal was to determine the parameter combinations predicted to maximize the simultaneous recovery of proteins, polyphenols, and flavonoids from *C. cornucopioides* using each extraction technique.

## 3. Results and Discussion

### 3.1. pH and Electrical Conductivity

The pH and electrical conductivity (*κ*) of the black trumpet extracts provide critical insights into their physicochemical properties and the efficiency of solute release during the extraction process. In this study, the pH values remained within a narrow, slightly acidic range of 5.51 to 5.88. This stability indicates that the applied extraction conditions, whether MAE or UAE, did not cause significant degradation of the fungal matrix into strongly acidic or alkaline by-products. A distinct differentiation was observed based on the solvent type; extracts prepared with distilled water exhibited consistently lower pH values (5.51–5.62) compared to those prepared with 30% ethanol, which reached values up to 5.88. This suggests that pure water more effectively facilitates the dissociation and recovery of acidic secondary metabolites and organic acids naturally present in the fruiting bodies of the black trumpet. Furthermore, this observed slightly acidic pH range may be partially attributed to the ecological characteristics of *C. cornucopioides*. This species typically thrives in deciduous forests on acidic soils, which can influence the accumulation of specific organic acids and ionizable metabolites within the fruiting bodies. The efficient recovery of these acidic constituents, particularly in aqueous media, reflects both the natural metabolic profile of the mushroom and the influence of its growth environment on the final chemical composition of the extracts [7].

Electrical conductivity serves as a robust metric for quantifying the total dissolved ionic content within the extracts, effectively reflecting the degree to which ionizable constituents, including mineral salts, organic acids, and certain nitrogenous compounds, are liberated from the complex fungal matrix [27]. The results revealed a twofold higher conductivity in the aqueous extracts compared to the 30% ethanol extracts. This confirms that water, as a highly polar solvent, is superior for the recovery of ionic constituents from this species. Within the UAE series, conductivity in the ethanolic extracts increased progressively with higher amplitudes and longer extraction times. This trend confirms that acoustic cavitation effectively disrupts the rigid chitinous cell walls, facilitating a more intensive mass transfer of intracellular electrolytes. Notably, the absolute highest conductivity was recorded in the aqueous microwave extract MW1, suggesting that rapid initial microwave heating provides an immediate and efficient release of water-soluble ions.

### 3.2. Phytochemical Composition: Total Phenolic Content (TPC) and Total Flavonoid Content (TFC)

The specific phytochemical profile of *C. cornucopioides* was characterized by measuring TPC and TFC, revealing that both the extraction technique and the solvent significantly impacted the recovery of bioactive compounds (Table 2). The TPC ranged from 3.52 to 6.06 mg GAE/g d.m., while the TFC showed greater variability, ranging from 1.26 to 5.79 mg QE/g d.m. Consistent with the trends observed for protein recovery, electrical conductivity, and antioxidant activity, UAE in an aqueous medium proved to be the most efficient method for the liberation of polyphenols. Sample UW4 (6 min, 100% amplitude) yielded the highest concentrations of both TPC (6.06 ± 0.18 mg GAE/g d.m.) and TFC (5.79 ± 0.28 mg QE/g d.m.). This quantitative characterization highlights the superiority of UAE, which can be attributed to the mechanical effects of acoustic cavitation, which creates micro-fractures in the fungal cell wall. Our results provide a robust baseline for the chemical fingerprint of these extracts, demonstrating that by utilizing water and high-amplitude ultrasound, it is possible to bridge the gap between “low-content” reports in the literature and the actual bioactive potential of the black trumpet recovered through our optimized protocol. In the case of flavonoids, the UAE–water series showed a nearly twofold increase in yield compared to the MAE series, suggesting that flavonoids in the black trumpet may be sensitive to the rapid localized heating associated with microwave radiation, or that they are more effectively solubilized through mechanical vibration. Regarding MAE, a clear temperature-dependent trend was observed. Increasing the temperature from 40 °C to 60 °C enhanced the TPC by approximately 13–15% in both water and ethanol extracts. This indicates that higher thermal energy reduces the viscosity of the solvent and improves the solubility of phenolic compounds from the complex fungal matrix. However, the MAE-EtOH series yielded lower values for all parameters compared to MAE–Water. This confirms that the investigated bioactive constituents of *C. cornucopioides* have high polarity, making water the optimal solvent for their recovery.

The results of this study, particularly the values obtained for the UW4 sample, demonstrate the high efficiency of UAE compared to traditional methods described in the literature. According to the comprehensive review by Adamska and Felisiak [8], the total phenolic content in *C. cornucopioides* typically exhibits significant variability depending on the extraction method and solvent. While several studies cited in the review report relatively low phenolic concentrations, ranging from 1.6 to 5 mg/g d.m. [28,29,30], our UW4 extract slightly exceeds these common benchmarks. This is consistent with the observation that aqueous extracts tend to yield significantly higher phenolic concentrations than methanolic ones, a trend also noted in previous reports where water extracts provided nearly double the yield [11]. Regarding the TFC, Adamska and Felisiak emphasize that *C. cornucopioides* is often characterized by modest concentrations, with some researchers reporting values as low as 1.71 mg/g d.m. [30] In contrast, our UW4 protocol achieved a substantially higher flavonoid recovery of 5.79 mg QE/g d.m. This discrepancy is likely due to the mechanical disruption of the fungal cell wall caused by acoustic cavitation, which allows for a more intensive release of bioactive compounds that are often “underestimated” in studies using conventional maceration. Furthermore, while some literature reports extreme values reaching up to 37.5 mg/g d.m. [9], such results are often considered outliers or highly dependent on specific geographic origins. Our results provide a robust and reproducible baseline, demonstrating that by utilizing water and high-amplitude ultrasound, it is possible to bridge the gap between “low-content” reports and the actual bioactive potential of the black trumpet. This confirms that the UW4 extraction condition represents a superior, eco-friendly approach for maximizing the recovery of both polyphenols and flavonoids from this species.

### 3.3. Evaluation of Total Protein Content

The specific chemical profile of the nitrogenous fraction in *C. cornucopioides* extracts was characterized using Bradford and Lowry assays, revealing significant insights into the nature of the compounds recovered under our unique MAE and UAE protocols (Table 2). This quantitative characterization showed a substantial difference between the results of the two assays; the Lowry method yielded values (32.06–55.57 mg BSA/g d.m.) that were consistently one order of magnitude higher than those obtained by the Bradford method (1.52–9.93 mg BSA/g d.m.). This discrepancy is inherently linked to the chemical principles of each assay. The Bradford reagent primarily binds to high-molecular-weight proteins, whereas the Lowry assay is sensitive to both proteins and smaller peptides, as well as non-proteinaceous reducing agents [31,32]. In the context of the black trumpet, the elevated Lowry values likely reflect a high concentration of short-chain peptides and certain phenolic compounds that can interfere with the Folin–Ciocalteu reagent. While Turfan et al. [9] reported general high amounts of free amino acids in this species, our results uniquely characterize how MAE and UAE selectively recover these nitrogenous markers. Since the Lowry assay is sensitive to these nitrogenous monomers and small peptides, their abundance in the black trumpet directly contributes to the higher protein values recorded compared to the Bradford assay. However, both methods followed an identical trend, confirming the reliability of the observed extraction patterns. The efficiency of protein liberation was significantly enhanced by UAE, with the aqueous extract UW4 achieving the highest protein recovery. This suggests that the intense shear forces and micro-jets generated by acoustic cavitation are particularly effective at deconstructing the chitin–glucan complex of the fungal cell wall. Therefore, the values reported in Table 2 serve as specific chemical markers for the efficiency of the physical disruption provided by our optimized methods. Furthermore, the higher protein yield in water-based extracts compared to 30% ethanol confirms that the majority of the proteins in *C. cornucopioides* are water-soluble albumins or polar glycoproteins. In the MAE series, samples treated at 60 °C (MW4 and ME4) showed a significant increase in protein content compared to those at 40 °C, confirming that thermal energy improves solvent penetration, although it remains less effective than the mechanical disruption provided by UAE.

The protein yields obtained in this study, specifically for the UW4 sample, align with the high nutritional value of *C. cornucopioides* reported in primary studies. According to Barros et al. [28], the fruiting bodies of the black trumpet are characterized by a significant protein fraction, although its accessibility is often limited by the complex fungal matrix. The high efficiency of our UW4 protocol is consistent with the findings of Ouali et al. [29], who emphasized that water-soluble nitrogenous compounds constitute a major portion of the bioactive pool in this species. Furthermore, the substantial values recorded in the Lowry assay in our study can be explained by the presence of specific peptides and free amino acids, such as those identified by Turfan et al. [9], which can cross-react in the assay. Therefore, while standard analyses report the total protein potential of the biomass [13], our optimized UW4 method specifically maximizes the yield of soluble proteins and peptides in the extract. This makes it a superior technique for the practical production of nutrient-dense fungal extracts where bioavailable nitrogenous compounds are a key target.

**Figure 1:**
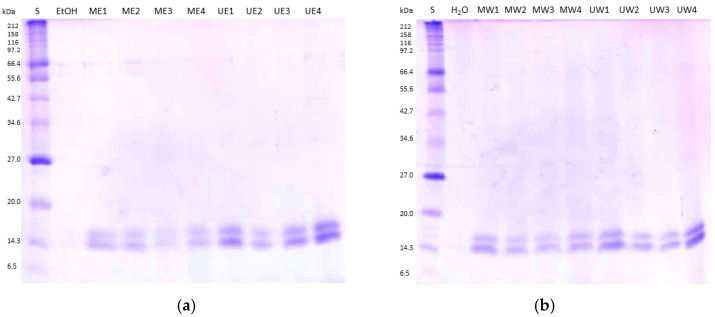
Protein profile of (**a**) ethanolic and (**b**) aqueous extracts obtained by SDS-PAGE.

The SDS-PAGE analysis further complemented the quantitative characterization by revealing the molecular weight distribution. The presence of distinct low-molecular-weight bands in our extracts confirms that the recovered chemical profile is rich in nitrogenous species that are readily detected by the Lowry method. This qualitative fingerprint, combined with the quantitative data, provides comprehensive insight into the proteinaceous composition unique to our optimized extraction conditions (Figure 1). Specifically, aqueous extracts showed two major bands at approximately 14.3 kDa, whereas ethanol extracts contained the same two bands in addition to two other bands ranging from 55.6 to 66.4 kDa, although these were faintly visible. These observations are not consistent with the quantitative Lowry and Bradford results, as ethanol extracts contain more protein bands than aqueous extracts. However, it should be noted that SDS-PAGE provides qualitative information on protein composition rather than precise concentrations; while band intensity can offer a rough indication of relative abundance, it cannot be directly equated to total protein content. The SDS-PAGE findings further elucidate the discrepancy between the two colorimetric assays; the presence of distinct low-molecular-weight bands and potential peptide fragments confirms that the extract is rich in nitrogenous species that are readily detected by the Lowry method but fall below the sensitivity threshold or binding requirements of the Bradford reagent.

### 3.4. Evaluation of Antioxidant Capacity

**Figure 2:**
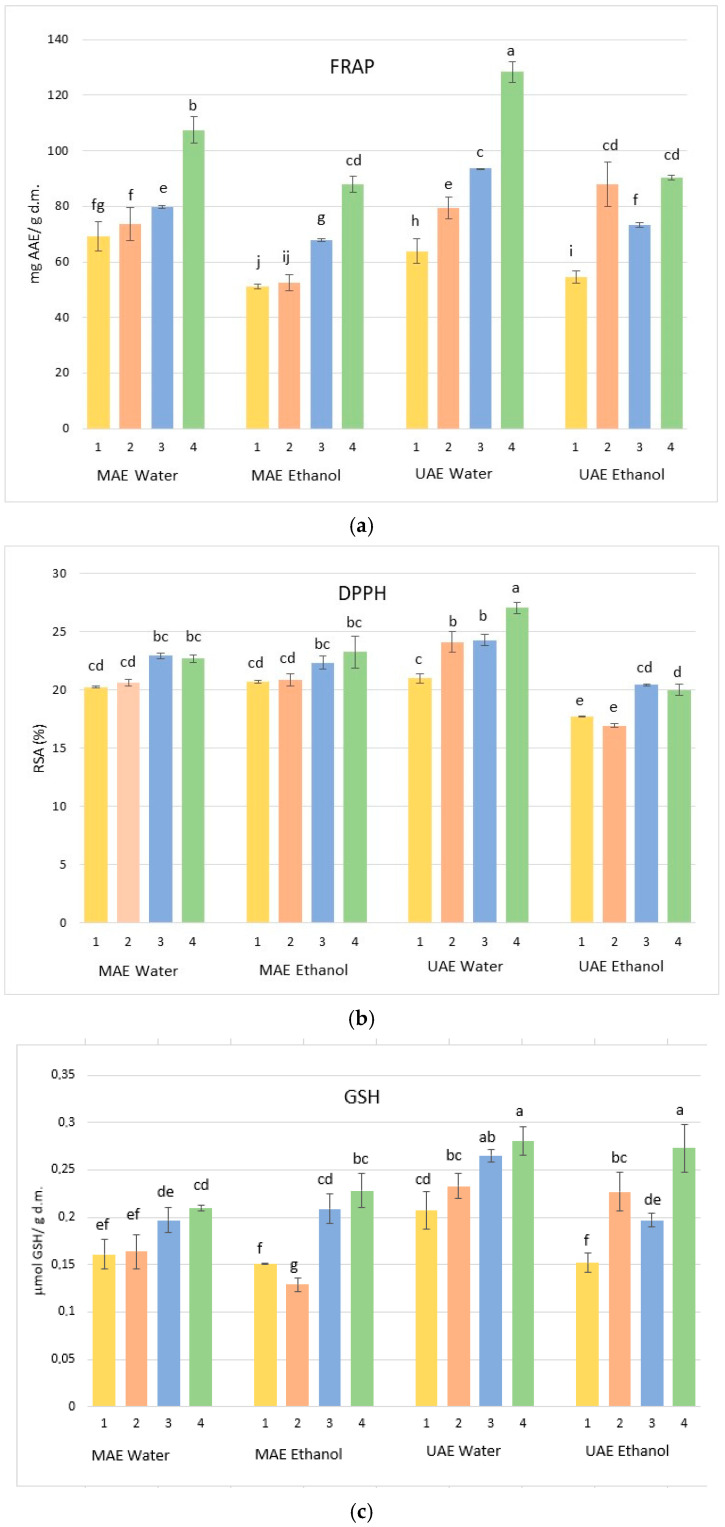
Comparative antioxidant capacity of *C. cornucopioides* extracts obtained by MAE and UAE in water and 30% ethanol: (**a**) Ferric Reducing Antioxidant Power (FRAP), (**b**) DPPH radical scavenging activity and (**c**) GSH concentration determined by Ellman’s DTNB assay. Values are expressed as mean ± standard deviation (*n* = 2). Different letters indicate statistically significant differences among extracts. The samples 1–4 correspond to those in Table 1.

The antioxidant potential of *C. cornucopioides* extracts was further elucidated through FRAP and DPPH assays, which assess the reducing power and radical scavenging activity, respectively. As illustrated in Figure 2a,b, a consistent trend was observed where the antioxidant capacity directly correlated with the concentration of phenolic and flavonoid compounds. The FRAP assay revealed a wide range of values, with the highest reducing power recorded for the aqueous ultrasound-assisted extract UW4, significantly outperforming the microwave-assisted equivalents. This suggests that the intensive mechanical effects of acoustic cavitation not only enhance the yield of bioactive molecules but also potentially preserve their redox-active structures more effectively than rapid microwave heating. Similarly, the DPPH radical scavenging activity followed a comparable pattern, with the maximum inhibition percentage achieved by the UW4 sample. Interestingly, while the MAE–water series showed relatively high FRAP values, their DPPH inhibition remained lower than that of the UAE–water series, indicating that ultrasound may be more effective at liberating specific flavonoid fractions with higher affinity for radical neutralization.

Conversely, the ME and UE extracts generally exhibited lower antioxidant performance, which aligns with their reduced TPC. Overall, these results confirm that the synergy between water as a solvent and high-amplitude ultrasound provides the most potent bioactive extracts from the black trumpet mushroom.

According to the data summarized by Adamska and Felisiak [8], the antioxidant activity of *C. cornucopioides* is highly variable and solvent-dependent. Our FRAP results show a remarkable improvement in reducing power compared to the aqueous extracts reported by Costea et al. [33], who recorded 17.39 ± 1.73 mg AAE/g. The nearly sevenfold increase in our UW4 sample suggests that high-amplitude ultrasound facilitates a much more intensive recovery of redox-active metabolites than simple aqueous maceration. This superior performance aligns with the trend observed by Radović et al. [11], where distilled water significantly outperformed ethanolic and methanolic solvents in ferric reducing power. Regarding radical scavenging activity, many authors cited in the review report relatively low DPPH potential, with EC_50_ values often exceeding 7.5 mg/mL [34] or reaching as high as 40 mg/mL [35]. In our study, the UW4 extract achieved 27.06% inhibition using only 100 µL of sample in a 2.6 mL reaction mixture. This robust inhibition under such high dilution indicates that the actual EC_50_ of our extract would be significantly lower, and thus more potent, than the values reported by Queirós et al. [34] and Vasdekis et al. [35]. This high scavenging efficiency is comparable to the “potent activity” observed by Kosanić et al. [10] in acetone extracts, but achieved here through an eco-friendly aqueous medium.

In addition to FRAP and DPPH assays, the antioxidant capacity of the extracts was further evaluated using Ellman’s DTNB assay, which reflects the presence of thiol-containing and redox-active compounds. The results (Figure 2c) demonstrated that antioxidant potential was also influenced by extraction conditions, although the pattern differed slightly from that observed in FRAP and DPPH analyses. Among MAE extracts, the highest activity was recorded for the aqueous samples MW3 and MW4, as well as for the ethanolic extracts ME3 and ME4. In the UAE series, aqueous extracts UW3 and UW4 exhibited the strongest activity, while UE4 and UE2 were the most active among the ethanolic UAE extracts. Overall, the most pronounced antioxidant response across all GSH measurements was observed for UW4, followed by UW3 and UE4. These findings partially corroborate the FRAP and DPPH results, confirming the superior performance of high-amplitude ultrasound in combination with water as a solvent. However, the DTNB assay highlights that certain ethanol-based extracts (notably UE4 and ME4) also possess considerable redox activity, suggesting the extraction of specific thiol-containing or low-molecular-weight antioxidant compounds that may not be fully reflected in ferric reducing power or radical scavenging assays. Despite the recognized importance of GSH as a key intracellular antioxidant, information regarding mushrooms as a direct dietary source of GSH remains limited. Dogan et al. [36] reported GSH concentrations ranging from 250 to 1600 μg/g fresh weight in eight edible mushroom species; however, *C. cornucopioides* was not included in their analysis. Therefore, the present results provide additional data on the glutathione content of this species and contribute to a more comprehensive characterization of its antioxidant profile.

The standardized chemical markers (TPC and TFC) and the antioxidant responses recorded here characterize our specific MAE and UAE products, rather than just reflecting general species potential. Furthermore, the glutathione (GSH) concentration was utilized as a specific molecular marker to differentiate the effects of microwave vs. ultrasound energy. Our results provide new data on the thiol-based antioxidant profile of *C. cornucopioides* and, in combination with TPC and TFC, establish a comprehensive chemical characterization of the optimized extracts.

### 3.5. Evaluation of Antimicrobial Activity

In the present study, a comprehensive antimicrobial screening of the optimized MAE and UAE extracts was performed against a wide range of pathogens, including *Proteus mirabilis*, *Escherichia coli*, *Bacillus subtilis*, *Pseudomonas aeruginosa*, *Staphylococcus aureus*, *Salmonella typhimurium*, *Listeria monocytogenes*, and *Candida albicans*. The evaluation was carried out using three complementary methods: disk diffusion, agar well diffusion, and turbidimetric assays, with a standardized cell density of 0.2 McFarland (approx. 10^6^ CFU/mL). Despite the extensive screening, the results showed that neither the ethanol nor the aqueous extracts prepared at a concentration of 30 mg/g exhibited detectable antimicrobial activity (no zones of inhibition or significant growth reduction in turbidimetric assays) against any of the tested strains under the conditions employed. Moreover, the concentration of the extract is a determining factor for antimicrobial effectiveness. Our results suggest that a concentration of 30 mg/g is likely insufficient to reach the minimum inhibitory concentration (MIC) required to suppress microbial growth across these diverse bacterial and fungal species in the tested assays. A previous study, however, reported that *C. cornucopioides* inhibited the growth of *S. aureus* when extracted with acetone, while the methanolic extract inhibited the growth of *K. pneumoniae*, though activity was apparent only at the highest tested concentration (200 mg/g) [37]. Similarly, Kol et al. [38] evaluated the antimicrobial activity of *C. cornucopioides* extracts prepared in methanol and water and concluded that methanolic extracts exhibited greater antimicrobial activity than aqueous extracts. These findings indicate that, in addition to extract concentration, the choice of solvent plays a critical role in determining antimicrobial efficacy.

The solvent influences antimicrobial activity by determining which bioactive compounds are extracted. Many of the most potent antimicrobial constituents of *C. cornucopioides*, including phenolic compounds and terpenoids, are more soluble in medium-polarity organic solvents. A 30% ethanol solution, due to its high water content, is relatively polar and therefore less efficient at extracting moderately polar or non-polar antimicrobial compounds, resulting in reduced biological activity. Water is also a limited extraction solvent in this context, as it primarily solubilizes highly polar compounds such as polysaccharides, whereas several of the most active antimicrobial molecules (e.g., phenolics and terpenoids) exhibit greater solubility in organic solvents such as ethanol or methanol [39]. Furthermore, the mechanical and physical effects of the extraction process itself, particularly in UAE, play a significant role in determining the overall antimicrobial potency of the system. According to Liu et al. [40], ultrasound stimulation can effectively loosen dense microbial structures and biofilms, promoting the irreversible destruction of cell walls and increasing the production of reactive oxygen species. This suggests that while the current crude extracts at 30 mg/g concentration did not reach the MIC in a static assay, the UAE process utilized in this study could potentially enhance the delivery and efficacy of these bioactives in more dynamic or high-intensity applications, such as active food packaging [40]. The broader potential of ultrasound-mediated biological activity is further highlighted by its ability to induce spatiotemporal biological responses. Beyond extraction, ultrasound-mediated mechanical and cavitation effects are known to trigger mitochondrial stress and synergistic cell death in targeted systems, such as advanced ultrasound-responsive nanomaterials. Integrating such advanced UAE principles into the development of bio-active packaging could lead to the creation of ‘smart’ materials where the antimicrobial action is further triggered or enhanced by external stimuli, aligning our optimization results with the next generation of eco-friendly and high-performance food contact materials [41].

Although methanol and acetone may enhance the extraction efficiency of antimicrobial compounds due to their strong solvating capacity for moderately polar and non-polar molecules, their use is not suitable for bio-packaging applications. Both solvents are toxic, highly volatile, and not food-grade, raising significant safety, regulatory, and environmental concerns, particularly for materials intended for food contact [42]. This is a crucial consideration, as the obtained extracts are primarily evaluated for their potential to be incorporated into bio-packaging matrices to develop active food contact materials in future applications*.* In addition, potential solvent residues could compromise consumer safety and negatively affect the biodegradability and sustainability profile of the potential packaging material. In contrast, water and ethanol represent safer and more sustainable alternatives. Ethanol is food-grade and environmentally acceptable, while water is non-toxic, inexpensive, and fully aligned with green extraction principles, making these solvents more appropriate for the development of eco-friendly antimicrobial bio-packaging systems.

### 3.6. Optimization of Extraction

In this study, the influence of extraction parameters on the recovery efficiency of key bioactive compounds from *C. cornucopioides* was investigated using two advanced techniques, MAE and UAE. For each method, a dual statistical approach was applied. First, a Multifactor Analysis of Variance was performed to determine the statistical significance of the main effects of the critical parameters: treatment time (TT) and solvent type (S) for both methods, with the third factor being temperature (T) for MAE and amplitude (A) for UAE. The significance of these main effects was evaluated using a main-effects-only model, which confirmed their statistical importance for most responses. Second, Response Surface Methodology (RSM) was employed to model the interactions between these factors and to identify conditions associated with maximum extraction yields within the studied experimental domain. The main statistical results for MAE and UAE are presented in Table 3, Table 4, Table 5 and Table 6, followed by a detailed discussion of the factor influence, model performance, and a comparative analysis of the two extraction techniques.

#### 3.6.1. Statistical Significance and Main Factor Influence in MAE

The Multifactor Analysis of Variance results (Table 3) reveal distinct patterns in how the MAE parameters influence different target compounds. Temperature (T) emerged as the most dominant and statistically significant factor for all responses (as shown in Table 3), including TPC (*p* = 0.0061), TFC (*p* = 0.0008), DPPH (*p* = 0.0007), FRAP (*p* = 0.0123), GSH (*p* = 0.0081) and protein yield measured by both Bradford (*p* = 0.0117) and Lowry (*p* = 0.0046) methods. This overwhelming influence underscores the primary role of thermal energy in MAE. The solvent type (S) was another highly significant factor (*p* < 0.05) for all parameters except DPPH (*p* = 0.5302) and GSH content (*p* = 0.7741). Consistently, aqueous extracts yielded significantly higher amounts of polyphenols, flavonoids, proteins, and demonstrated greater ferric reducing power compared to ethanolic extracts. This can be attributed to the polar nature of water, which is highly effective at dissolving a wide range of polar and mid-polar compounds, including many phenolic acids, sugars, and proteins present in mushrooms. Ethanol, while a good solvent for less polar flavonoids and certain antioxidants, may have been less efficient in disrupting the fungal cell wall and extracting the broader spectrum of compounds quantified in these assays. Interestingly, GSH recovery was uniquely influenced only by temperature, while solvent type and extraction time did not show statistical significance. This suggests that the liberation of glutathione from the fungal matrix is highly thermo-dependent, but its stability or solubility under these specific MAE conditions is less affected by the ethanol–water ratio compared to other bioactive classes. For the recovery of bioactive compounds, the influence of treatment time (TT) was not significant, suggesting that the rapid heating characteristic of MAE leads to efficient extraction quickly, and prolonging the exposure time does not substantially increase yields.

**Table 3:** Multifactor analysis of variance for bioactive compounds and antioxidant capacity.

Response	Factor	Sum of Squares	Df	Mean Square	*F*-Ratio	*p*-Value
Proteins (Bradford)	TT	0.6172	1	0.6172	4.45	0.1026
	T	2.6912	1	2.6912	19.40	0.0117
	S	2.5268	1	2.5268	18.21	0.0130
TPC	TT	0.0611	1	0.0611	3.14	0.1513
	T	0.5486	1	0.5486	28.17	0.0061
	S	0.4555	1	0.4555	23.39	0.0084
TFC	TT	0.0104	1	0.0104	1.04	0.3650
	T	0.8405	1	0.8405	83.90	0.0008
	S	0.2162	1	0.2162	21.58	0.0097
DPPH	TT	0.1947	1	0.1947	1.87	0.2430
	T	9.5528	1	9.5528	91.87	0.0007
	S	0.0490	1	0.0490	0.47	0.5302
FRAP	TT	358.37	1	358.37	5.79	0.0739
	T	1165.04	1	1165.04	18.81	0.0123
	S	623.82	1	623.82	10.07	0.0337
GSH	TT	2.1125·10^−5^	1	2.1125·10^−5^	0.07	0.8033
	T	7.14012·10^−3^	1	7.14012·10^−3^	23.93	0.0081
	S	2.8125·10^−5^	1	2.8125·10^−5^	0.09	0.7741
Proteins (Lowry)	TT	12.9719	1	12.9719	3.77	0.1241
	T	113.259	1	113.259	32.93	0.0046
	S	251.609	1	251.609	73.15	0.0010

#### 3.6.2. Modeling Interactions and Identification of Optimal Conditions for MAE via RSM

To move beyond assessing main effects and to understand the complex interplay between factors, RSM was applied. This approach allows for the creation of predictive mathematical models that describe how the response variables change across the experimental domain, including two-factor interactions. The results of the RSM analysis, including model fit, regression equations, and indicated optimal conditions, are summarized in Table 4. All fitted models exhibited excellent explanatory power, with high *R*-squared (>97.5%) and adjusted *R*-squared values (>82.8%), indicating a very good fit to the experimental data. The regression equations unveiled important two-factor interactions that the multifactor analysis of variance could not fully capture. For instance, in the TPC model, the positive coefficient for the TT·S interaction (+0.0602) indicates a synergistic effect where the combination of longer extraction time and water as the solvent was particularly beneficial for maximizing phenolic yield. Conversely, for FRAP, the strongly negative coefficient for the main effect of solvent (−19.46) and the TT·S interaction term (−1.771) emphasizes the clear superiority of water, an effect that was slightly more pronounced at shorter extraction times. The GSH model also revealed interesting dynamics; although the main effect of temperature was dominant, as shown in the multifactor ANOVA, the RSM model identified a slightly different optimal solvent composition (30% EtOH) compared to the pure aqueous preference observed for TPC and proteins. This shift, coupled with the negative coefficient for the TT × S interaction, suggests that while glutathione is readily extracted, its maximum recovery requires a delicate balance between solvent polarity and thermal exposure to prevent potential degradation or to optimize its solubility profile within the fungal matrix.

**Table 4:** Response Surface Methodology (RSM) models and optimization results for MAE.

Response	Model Fit (*R*^2^, adj. *R*^2^)	Regression Equation (Fitted Model)	Optimal Conditions for Maximum Response
TPC	0.9987, 0.9906	TPC = 3.896 − 0.136·TT + 0.0186·T − 0.0343·S + 0.00328·TT·T + 0.0602·TT·S − 0.0143·T·S	TT = 6 min, T = 60 °C, S = Water(0), predicted = 5.38 mg GAE/g
TFC	0.9994, 0.9958	TFC = −0.779 + 0.195·TT + 0.0528·T + 0.101·S − 0.00349·TT·T + 0.0075·TT·S − 0.00928·T·S	TT = 3 min, T = 60 °C, S = Water(0), predicted = 2.34 mg QE/g
DPPH	0.9755, 0.8282	DPPH = 15.857 − 0.022·TT + 0.113·T + 0.335·S + 0.001·TT·T + 0.152·TT·S − 0.0173·T·S	TT = 6 min, T = 60 °C, S = Ethanol(1), predicted = 23.1%
FRAP	0.9989, 0.9921	FRAP = 82.233 − 12.264·TT − 0.476·T − 19.462·S + 0.352·TT·T − 1.771·TT·S + 0.195·T·S	TT = 6 min, T = 60 °C, S = Water(0), predicted = 106.9 mg AAE/g
GSH	0.9857, 0.8997	GSH = 0.164125 − 0.0185833·TT+ 0.0001375·T − 0.08325·S + 0.000425·TT·T − 0.00316667·TT·S + 0.001875·T·S	TT = 6 min, T = 60 °C, S = 30% EtOH(1), predicted = 0.224125 μmol GSH/g
Proteins (Bradford)	0.9986, 0.99	Prot = 2.596 − 0.280·TT − 0.00748·T − 2.044·S + 0.0109·TT·T − 0.161·TT·S + 0.0329·T·S	TT = 6 min, T = 60 °C, S = Water(0), predicted = 4.39 mg BSA/g
Proteins (Lowry)	0.9981, 0.9865	Prot = 29.872 − 1.286·TT + 0.211·T + 0.121·S + 0.0523·TT·T − 0.959·TT·S − 0.140·T·S	TT = 6 min, T = 60 °C, S = Water(0), predicted = 53.7 mg BSA/g

Analysis of the model-indicated optima (Table 4, last column) identified the specific combination of factors within the studied range that is associated with maximizing each individual response. A dominant trend is observed: for the majority of responses, TPC, FRAP, and both protein assays, conditions indicated as optimal were 6 min, 60 °C, and water as a solvent. The exceptions were flavonoids, where a shorter time (3 min) was indicated; glutathione (GSH), which showed an optimum with 30% ethanol; and DPPH activity, which was marginally higher with ethanol. The model-indicated maximum for TPC (5.38 mg GAE/g) closely aligns with the experimentally observed maximum (5.36 mg GAE/g for sample MW4), demonstrating the model’s accuracy in describing the experimental data.

In conclusion, this study confirms that microwave-assisted extraction, guided by statistical experimental design, is a potent tool for recovering bioactive compounds from *C. cornucopioides*. Temperature was the most critical parameter, and water proved to be a more efficient solvent than ethanol for most target compounds. The derived RSM models, which showed excellent fit to the data (*R*^2^ > 0.97), are valuable for understanding factor interactions and identifying promising conditions for process tuning.

#### 3.6.3. Statistical Significance and Main Factor Influence in UAE

A parallel statistical investigation was conducted for UAE. The influence of treatment time (TT), amplitude (A), and solvent type (S) on the recovery of bioactive compounds was analyzed using multifactor analysis of variance and RSM. The results, summarized in Table 5 and Table 6, reveal a distinct factor significance profile compared to MAE. The results for UAE (Table 5) highlight a different dynamic. While solvent type (S) remained a critically significant factor (*p* < 0.05) for most responses, especially proteins (Bradford: *p* = 0.0015; Lowry: *p* = 0.0076) and polyphenols (TPC: *p* = 0.0119), the mechanical parameter amplitude (A) emerged as equally or more significant than time for many compounds.

Amplitude had a highly significant effect on TFC (*p* = 0.0292), DPPH activity (*p* = 0.0424), and both protein assays (Bradford: *p* = 0.0090; Lowry: *p* = 0.0180). This underscores the primary role of ultrasonic cavitation in UAE: higher amplitudes generate more intense cavitation bubbles, leading to greater cell wall disruption and improved mass transfer of intracellular compounds. Treatment time (TT) was a significant factor for DPPH (*p* = 0.0410) and showed borderline significance for TPC (*p* = 0.0526), FRAP (*p* = 0.0536) and proteins (Bradford: *p* = 0.0550). Specifically, for GSH recovery, both treatment time (*p* = 0.0249) and amplitude (*p* = 0.0266) were found to be statistically significant drivers, while the solvent type did not significantly influence the yield (*p* = 0.0721). This suggests that the release of glutathione in the UAE system is primarily governed by the physical effects of acoustic cavitation—where both the intensity (amplitude) and the duration of exposure facilitate the rupture of fungal structures—rather than the chemical affinity of the solvent. This indicates that, unlike in MAE, the duration of ultrasonic exposure plays a measurable role in the extraction yield, likely because the cavitation effect is cumulative over time. As with MAE, water was consistently a more effective solvent than ethanol for extracting polyphenols, flavonoids, and proteins. However, its effect on DPPH activity (*p* = 0.1568) was not significant in the UAE system. This is reflected in the multifactor ANOVA, where the solvent factor alone was not statistically significant for DPPH. However, the subsequent RSM model, which accounts for interactions, indicated a complex relationship where the combination of solvent with other parameters (notably time) influenced the outcome, ultimately pointing to water as part of the indicated optimum set of conditions.

**Table 5:** Multifactor analysis of variance for bioactive compounds and antioxidant capacity.

Response	Factor	Sum of Squares	Df	Mean Square	*F*-Ratio	*p*-Value
Proteins (Bradford)	TT	0.4935	1	0.4935	7.20	0.0550
	A	1.5409	1	1.5409	22.49	0.0090
	S	4.0684	1	4.0684	59.39	0.0015
TPC	TT	2.1914	1	2.1914	7.44	0.0526
	A	3.5126	1	3.5126	11.92	0.0260
	S	5.6566	1	5.6566	19.20	0.0119
TFC	TT	2.6370	1	2.6370	1.63	0.2710
	A	17.9311	1	17.9311	11.07	0.0292
	S	56.7698	1	56.7698	35.06	0.0041
DPPH	TT	1269.37	1	1269.37	8.84	0.0410
	A	1241.22	1	1241.22	8.65	0.0424
	S	434.80	1	434.80	3.03	0.1568
FRAP	TT	5.6583	1	5.6583	7.34	0.0536
	A	9.0525	1	9.0525	11.74	0.0266
	S	30.0235	1	30.0235	38.95	0.0034
GSH	TT	4.802·10^−3^	1	4.802·10^−3^	12.25	0.0249
	A	4.608·10^−3^	1	4.608·10^−3^	11.76	0.0266
	S	2.312·10^−3^	1	2.312·10^−3^	5.90	0.0721
Proteins (Lowry)	TT	45.649	1	45.649	5.69	0.0755
	A	119.955	1	119.955	14.96	0.0180
	S	198.403	1	198.403	24.75	0.0076

#### 3.6.4. Modeling and Identification of Optimal Conditions for UAE via RSM

The RSM models for UAE (Table 6) also demonstrated very high goodness-of-fit (*R*^2^ > 95.5% for all models), allowing for the identification of conditions associated with maximum yields within the experimental domain. The regression equations for UAE further elucidate the mechanical nature of this extraction method. For instance, in the GSH model, the positive interaction between treatment time and solvent indicates that the prolonged exposure to ultrasonic waves in an aqueous medium is particularly effective for glutathione recovery. This is consistent with the results for TPC, suggesting a unified mechanism where water not only acts as a superior solvent but also potentially enhances the transmission of ultrasonic energy and cavitation intensity compared to ethanol.

**Table 6:** Response Surface Methodology (RSM) models and optimization results for UAE.

Response	Model Fit (*R*^2^, adj. *R*^2^)	Regression Equation (Fitted Model)	Optimal Conditions for Maximum Response
TPC	0.9790, 0.8530	TPC = 3.888 − 0.0094·TT + 0.0136·A − 1.344·S + 0.00166·TT·A + 0.1005·TT·S − 0.00713·A·S	TT = 6 min, A = 100%, S = Water(0), predicted = 6.19 mg GAE/g
TFC	0.9970, 0.9791	TFC = −0.626 + 0.390·TT + 0.0409·A + 0.970·S + 0.00009·TT·A − 0.0948·TT·S − 0.0297·A·S	TT = 6 min, A = 100%, S = Water(0), predicted = 5.86 mg QE/g
DPPH	0.9992, 0.9946	DPPH = 15.127 + 0.965·TT + 0.0613·A + 0.365·S + 0.000183·TT·A − 1.192·TT·S − 0.00441·A·S	TT = 6 min, A = 100%, S = Water(0), predicted = 27.15%
FRAP	0.9555, 0.6885	FRAP = −2.439 + 7.722·TT + 0.747·A + 28.474·S + 0.00888·TT·A + 0.0197·TT·S − 0.577·A·S	TT = 6 min, A = 100%, S = Water(0), predicted = 123.92 mg AAE/g
GSH	0.9986, 0.9905	GSH = 0.1255 − 0.009333·TT+ 0.0012·A − 0.106·S − 0.0000333·TT·A + 0.01833·TT·S − 0.00014·A·S	TT = 6 min, A = 100%, S = Water(0), predicted = 0.2815 μmol GSH/g
Proteins (Bradford)	0.9965, 0.9757	Prot = −2.237 + 1.171·TT + 0.0965·A − 0.114·S − 0.00727·TT·A − 0.129·TT·S − 0.0424·A·S	TT = 6 min, A = 100%, S = Water(0), predicted = 10.07 mg BSA/g
Proteins (Lowry)	0.9999, 0.9994	Prot = 22.4385 + 1.587·TT + 0.228·A + 2.913·S + 0.00142·TT·A − 0.2013·TT·S − 0.1596·A·S	TT = 6 min, A = 100%, S = Water(0), predicted = 55.64 mg BSA/g

The analysis of model-indicated optima reveals a remarkably consistent trend: for all bioactive compounds and antioxidant activities, the conditions indicated as optimal for UAE were 6 min, 100% amplitude, and water. This unified indicated optimum simplifies the process design for maximizing the recovery of a broad spectrum of valuable compounds from *C. cornucopioides* using UAE. The model-indicated maximum for TPC (6.19 mg GAE/g) aligns very closely with the highest experimentally observed value (6.06 mg GAE/g for sample UW4).

#### 3.6.5. Comparative Remarks on MAE vs. UAE

**Figure 3:**
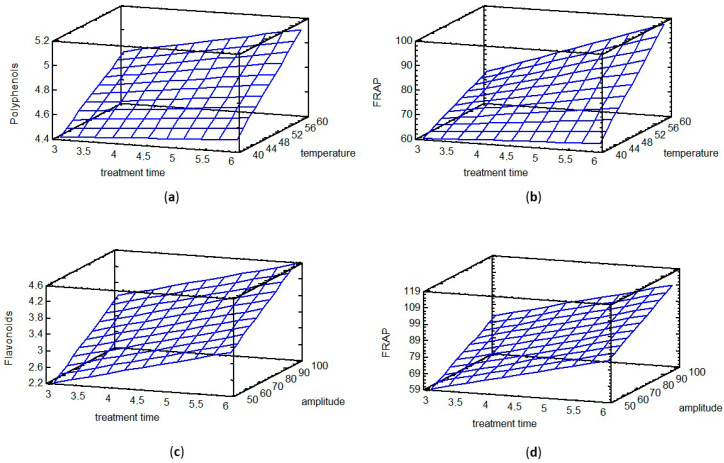
Response surface plots showing the effect of MAE temperature and treatment time on (**a**) TPC and (**b**) FRAP; the effect of UAE amplitude and treatment time on (**c**) TFC and (**d**) FRAP.

As visually summarized in Figure 3, the statistical comparison of both extraction techniques reveals their distinct operative principles and practical implications. The selected response surfaces represent the best-fitted quadratic models for key bioactive responses, illustrating the interactions between factors. The dominant factor diverged significantly between the methods. In MAE (Figure 3a,b), temperature was the overarching significant driver for compound recovery, highlighting the critical role of thermal energy in microwave-induced cell disruption. Specifically, Figure 3a demonstrates that maximum TPC requires both high temperature and extended time, as indicated by the positive interaction term in the model. While the surfaces in MAE appear relatively progressive, they confirm the necessity of reaching specific thermal thresholds for optimal recovery. Conversely, in UAE (Figure 3c,d), the mechanical parameter amplitude—which governs cavitation intensity—emerged as the primary significant factor for bioactive recovery. The response surfaces for TFC (Figure 3c) and FRAP (Figure 3d) exhibit a progressive increase, illustrating that extraction yields benefit consistently from the synergistic effect of maximum amplitude and extended treatment time. This visual consistency, where both phenolic compounds and antioxidant capacity follow a similar trend, suggests that UAE is a more robust and predictable process for the simultaneous recovery of multiple bioactive classes compared to MAE.

Regarding solvent efficacy, water proved superior in both systems, yet its effect was markedly more pronounced in UAE. In terms of process efficiency, UAE demonstrated a notable advantage by presenting a single, unified indicated optimum (6 min, 100% amplitude, water) for all target responses. This consistency, evident in the similar shape and predicted optimum location of the UAE surfaces in Figure 3c,d, suggests UAE is a more straightforward process for the simultaneous recovery of multiple bioactive compounds. MAE exhibited slightly different trends for different classes, as seen in the varying slopes for TPC versus the antioxidant capacity measured by FRAP (Figure 3b).

Qualitatively, although UAE and MAE equipment require significant electrical power during operation, their overall energy consumption is lower compared to traditional solid–liquid extractions (e.g., Soxhlet). This is attributed to the drastic reduction in extraction time—from several hours to a few minutes—and lower solvent usage, which minimizes the energy required for both the process and subsequent solvent recovery. In conclusion, UAE establishes itself as a highly effective and consistent method, with the cavitational mechanism, driven by amplitude, being decisive for the extraction of bioactives from *C. cornucopioides.*

#### 3.6.6. Global Process Optimization

To evaluate the overall efficiency of the extraction processes, a multi-response optimization was conducted using the desirability function (*D*). This statistical tool identifies the conditions where all studied responses reach their maximum simultaneous efficiency. For the UAE system, a near-perfect global desirability score of *D* = 0.98 was obtained at the unified conditions of 6 min, 100% amplitude, and water as the solvent. Similarly, the MAE system showed a high global desirability of *D* = 0.92 at 6 min, 60 °C, and water. These high *D* values, being close to 1.0, statistically confirm that the chosen optimal conditions are robust and effective for the balanced recovery of the entire bioactive profile of *C*. *cornucopioides*.

#### 3.6.7. Limitations and Outlook

A key methodological consideration of this optimization study is the absence of external validation experiments for the RSM models. While the models exhibit an excellent fit to the data, their true predictive accuracy for new conditions remains unconfirmed. Therefore, the ‘optimal conditions’ identified here are more accurately described as the best-performing parameter sets within the specific 2^3^ factorial design that was executed. They serve as a highly reliable guide for process development within these boundaries. Future work should include confirmatory runs at these indicated optima. Furthermore, exploring a broader factor space and conducting a formal multi-objective optimization balancing yield with energy efficiency would be valuable next steps for sustainable process development.

## 4. Conclusions

This study demonstrates that both MAE and UAE are highly effective, “green” methodologies for recovering bioactive compounds and proteins from the edible mushroom *C. cornucopioides*. Through RSM optimization, we established that temperature and solvent type are the primary drivers in MAE, while amplitude and treatment time govern the efficiency of UAE via mechanical cavitation. A key finding of this research is the consistent superiority of water as a solvent for extracting polyphenols, flavonoids, and proteins, aligning with sustainable and food-safe extraction principles. While MAE proved to be a rapid technique, UAE exhibited a more robust and predictable performance, presenting a unified indicated optimum (6 min, 100% amplitude, water) for all target responses. Notably, the extraction of glutathione (GSH) revealed distinct mechanistic pathways between the two methods: being strictly thermo-dependent in MAE, whereas in UAE, it was driven by the synergistic effect of amplitude and time. Furthermore, the higher protein yields observed in UAE, particularly those quantified by the Lowry method, suggest that ultrasonic cavitation facilitates a more intensive disruption of the fungal matrix, potentially leading to the release of smaller peptide fragments. Regarding the practical implementation and stability of the obtained extracts, the high concentration of bioactive proteins and phenolics suggests that these aqueous extracts should be stored under refrigerated conditions and protected from light to maintain their antioxidant potency and prevent degradation over time. In summary, the optimized aqueous extracts of *C. cornucopioides* obtained via UAE provide a superior profile of bioactive compounds and proteins. These findings establish a solid foundation for the future incorporation of these natural, safe, and sustainably sourced extracts into functional food products and active bio-packaging systems.

Study Limitations and Future Directions: While this study establishes optimized green extraction protocols for *C. cornucopioides*, it is limited to the quantification of broad bioactive classes and initial antimicrobial screening. Specific molecular characterization and advanced in vitro assays were beyond the current methodological scope, which focused on process optimization. However, as part of the project “Evaluation of alternative sources of bioactive compounds and proteins (BioAlter)”, the most potent extracts from this study have already been selected for the next research phase. This upcoming study will focus on protein glycation inhibition using HSA models and will include detailed HPLC-MS profiling to correlate specific metabolites with their therapeutic potential, providing the mechanistic depth that exceeds the scope of the present technological optimization.

## Figures and Tables

**Table 1:** Extraction conditions, pH, conductivity, temperature and energy consumption of microwave- and ultrasound-treated samples.

Sample Name	Treatment Time (min)	Amplitude (%)	Temperature (°C)	pH	*k* (mS cm^−1^)	*T* Initial/Final (°C)
MW1	3		40	5.56 ± 0.0	4.535 ± 0.007	20/41
MW2	6		40	5.615 ± 0.007	4.215 ± 0.007	20/42
MW3	3		60	5.525 ± 0.021	4.31 ± 0.0	20/63
MW4	6		60	5.510 ± 0.014	4.365 ± 0.007	20/61
ME1	3		40	5.87 ± 0.0	2.055 ± 0.007	20/42
ME2	6		40	5.87 ± 0.01	2.010 ± 0.014	20/44
ME3	3		60	5.83 ± 0.0	2.115 ± 0.007	20/62
ME4	6		60	5.825 ± 0.007	2.17 ± 0.0	20/61
UW1	3	50		5.60 ± 0.0	4.31 ± 0.0	14/21
UW2	6	50		5.60 ± 0.0	4.18 ± 0.0	16/25
UW3	3	100		5.565 ± 0.021	4.245 ± 0.007	16/33
UW4	6	100		5.595 ± 0.007	4.23 ± 0.014	18/33
UE1	3	50		5.880 ± 0.014	1.842 ± 0.001	16/26
UE2	6	50		5.865 ± 0.021	1.875 ± 0.001	20/23
UE3	3	100		5.885 ± 0.007	1.8955 ± 0.0035	13/29
UE4	6	100		5.88 ± 0.0	1.947 ± 0.004	18/35

**Table 2:** Total phenolic content (TPC), total flavonoid content (TFC), and total protein content (Bradford and Lowry assays) of *C. cornucopioides* extracts obtained by different extraction techniques and solvents.

Method	Sample	TPC (mg GAE/g d.m.)	TFC (mg QE/g d.m.)	Bradford Assay (mg BSA/g d.m.)	Lowry Assay (mg BSA/g d.m.)
MAE (water)	MW1	4.61 ± 0.20	1.49 ± 0.02	2.80 ± 0.09	41.04 ± 0.61
	MW2	4.63 ± 0.20	1.67 ± 0.16	3.20 ± 0.05	42.84 ± 0.39
	MW3	5.21 ± 0.10	2.35 ± 0.05	3.24 ± 0.17	47.79 ± 0.92
	MW4	5.36 ± 0.17	2.29 ± 0.09	4.43 ± 0.24	53.96 ± 0.11
MAE (30% EtOH)	ME1	4.22 ± 0.07	1.26 ± 0.00	1.52 ± 0.12	32.06 ± 0.33
	ME2	4.35 ± 0.10	1.43 ± 0.05	1.58 ± 0.01	32.21 ± 0.31
	ME3	4.47 ± 0.32	1.90 ± 0.04	2.75 ± 0.27	37.22 ± 0.44
	ME4	4.86 ± 0.04	1.90 ± 0.01	3.32 ± 0.25	39.28 ± 0.63
UAE (water)	UW1	4.66 ± 0.20	2.53 ± 0.00	4.86 ± 0.20	38.76 ± 1.75
	UW2	5.14 ± 0.02	3.85 ± 0.14	7.57 ± 0.08	43.87 ± 0.10
	UW3	5.85 ± 0.01	4.73 ± 0.22	8.89 ± 0.59	50.52 ± 1.14
	UW4	6.06 ± 0.18	5.79 ± 0.28	9.93 ± 0.99	55.57 ± 0.03
UAE (30% EtOH)	UE1	3.52 ± 0.22	1.87 ± 0.04	2.53 ± 0.29	33.22 ± 0.31
	UE2	3.79 ± 0.17	2.63 ± 0.08	4.28 ± 0.31	37.46 ± 0.64
	UE3	3.84 ± 0.11	2.31 ± 0.12	3.86 ± 0.23	36.74 ± 1.92
	UE4	4.87 ± 0.30	3.36 ± 0.07	5.09 ± 0.09	41.45 ± 1.26

Values are expressed as mean ± standard deviation (*n* = 2).

## Data Availability

The original contributions presented in this study are included in the article. Further inquiries can be directed to the corresponding author.
